# Clinical characteristics and treatment outcome in p16 negative anal cancer

**DOI:** 10.2340/1651-226X.2025.42498

**Published:** 2025-05-07

**Authors:** Catherine Burgos, Calin Radu, Sofia Heyman, Nina Cavalli-Björkman, Peter Nygren

**Affiliations:** a Department of Immunology, Genetics and Pathology, Uppsala University, Uppsala, Sweden; b Department of Oncology, Institute of Clinical Sciences, Sahlgrenska Academy, University of Gothenburg, Gothenburg, Sweden

**Keywords:** Anal squamous cell carcinoma, p16, clinical characteristics, prognosis

## Abstract

**Background:**

Anal squamous cell carcinoma (ASCC) is linked to human papillomavirus infection with p16 being positive in about 85% of cases. Overall survival (OS) of ASCC is 60%–80%. Prognosis in p16 negative (p16-) ASCC is worse with an OS of 30%–60%. It is important to elucidate differences in p16+ and p16- ASCC characteristics and outcome.

**Methods:**

Consecutive ASCC patients (*n* = 380) treated with curative intent in Uppsala 2017–2022 were reviewed and analyzed retrospectively. A cohort of p16- patients (*n* = 30) from Gothenburg was included as a validation cohort.

**Results:**

Ninety-one per cent (*n* = 347) were p16+ and 9% (*n* = 33) p16-. Median follow-up was 33 months (range 4–78). p16- status was associated with higher age (≥65 years; *p* = 0.03), comorbidity (*p* = 0.03), male sex (*p* = 0.001) and perianal localization (*p* < 0.001). At 3 years progression free survival was 50% and 81% (*p* <0.0001) and OS 60% and 89% (*p* < 0.0001) for p16- and p16+ patients, respectively. Male sex, advanced T-stage (T3-4), N+ disease, advance treatment and p16- status were associated with inferior OS (*p* = 0.01 – *p* < 0.0001). In the p16- subgroup, advanced T-stage and intensive treatment were negative prognostic factors for OS (*p* = 0.007 and 0.009, respectively) but no clinical characteristic predicted persistent disease. The p16- validation cohort essentially confirmed the findings from the main cohort.

**Interpretation:**

p16- ASCC is a disease subset with specific clinical features and poor prognosis in need of improved treatment.

## Introduction

Anal squamous cell carcinoma (ASCC) is an uncommon cancer diagnosis increasing in incidence worldwide [1, 2]. ASCC is strongly associated with human papillomavirus (HPV) infection particularly HPV types 16 and 18 [3–5], contributing to more than 80%–95% of ASCC cases [6–10]. Expression of p16 (p16+) is a well-established surrogate marker of HPV association in squamous cell cancers, including ASCC [11, 12] although discordant expression is well established [13–15]. In ASCC, 5%–20% of all tumors are reported p16 negative (p16-) [16–18]. This represents a small subset of ASCC patients, difficult to gain clinical experience and knowledge from.

p16- ASCC has been associated with unfavorable prognosis across multiple retrospective studies [17, 19–26]. Three meta-analyses have been published within this field [13–15] concluding that patients with p16- ASCC have poor prognosis with shortened overall survival (OS) compared with patients with p16+ ASCC.

The low prevalence of p16- ASCC, diversity in treatment and insufficient data on demographic and clinical characteristics, as reported, contribute to an incomplete picture of p16- ASCC. Hence, a retrospective analysis of ASCC-patients treated in accordance with the national care program was conducted to compare p16- and p16+ ASCC concerning clinical features, treatment outcome and prognosis.

## Material and methods

### Study population

All patients diagnosed with confirmed ASCC who underwent treatment with chemoradiotherapy (CRT) or radiotherapy (RT) with curative intent in accordance with the Swedish national care program at the Department of Oncology, Uppsala University Hospital, 2017–2022, were included in this retrospective cohort study. Patients with lymph node involvement extending to the common iliac artery or beyond were included if curative treatment with extended RT fields could be performed [27, 28]. Diagnostic procedures included digital examination, biopsy and pathology assessment, magnetic resonance imaging (MRI) and [18F]-fluorodeoxyglucose (FDG) positron-emission tomography combined with computerized tomography (PET-CT) [29, 30]. Tumor staging was performed according to the UICC TNM system 7th and 8th edition [31]. p16 status was determined by immunohistochemistry [32, 33].

Treatment was standardized in accordance with the national care program for ASCC as outlined in Appendix Table 1. Treatment protocols followed the Nordic Anal Cancer (NOAC) group and UK Co-ordinating Committee on Cancer Research (UKCCCR) guidelines [34]. Volumetric modulated arc therapy (VMAT) with simultaneous integrated boost (SIB) was used. RT was administered with doses ranging 44–58 Gy (2–2.13 Gy/22–27 fractions), 5 fractions per week to the tumor and involved lymph nodes (N+), while 40.7–41.6 Gy (1.85–1.54 Gy/22–27 fractions), 5 fractions per week was delivered to the elective lymph node regions. Five patients (all p16+) had identical RT but with protons instead of photons within the SWANCA study (NCT04462042). Chemotherapy combined with RT was Capecitabine (Cap)/5-Fluorouracil (5-FU) with one or two cycles of Mitomycin (MMC). CRT was adapted to tumor and node status and was administered according to schedule A: one cycle of MMC combined with Capecitabine/5-FU and a tumor dose of 44 Gy, schedule B: same chemotherapy with 54 Gy to the tumor or schedule C: two cycles of MMC combined with Capecitabine/5-FU and a tumor dose of 58 Gy. For patients with contraindications to Cap, weekly Cisplatin was used as an alternative. Patients treated with RT alone received doses ranging 61–64 Gy (2.13 Gy/30–32 fractions).

Data regarding tumor stage and characteristics, chemotherapy, oncologic outcome, and survival were retrieved from medical charts. Comorbidity was calculated according to the Charlson Comorbidity Index (CCI) [35]. Clinical complete response (cCR) was defined as no evidence of remaining tumor based on MRI, PET-CT and clinical examination at follow-up 6 months after end of treatment. Progression free survival (PFS) was calculated as time from the date of diagnosis to the finding of persistent tumor at 6 months follow-up, locoregional or distant relapse or death from any cause. OS and disease specific survival (DSS) were calculated as time from the date of diagnosis to date of death from any cause or due to ASCC, respectively. Patient characteristics according to p16 status are detailed in Table 1.

**Table 1:** Patient characteristics according to cohort and p16-status.

Characteristics	p16+ Uppsala (*n* = 347)	p16- Uppsala (*n* = 33)	*P*	p16- Gothenburg (*n* = 30)
**Age (mean; range)**	67 (29–89)	71 (40–83)		70 (37–92)
	**No (%)**	**No (%)**		**No (%)**
< 65 years	162 (47)	9 (27)	**0.03**	10 (33)
≥ 65 years	185 (53)	24 (73)		20 (67)
**Sex**				
Male	74 (21)	17 (52)	**0.0001**	21(70)
Female	273 (79)	16 (48)		9 (30)
**Smoking**				
Former/current	222 (64)	25 (76)	0.18	13 (43)
Non-smoker	125 (36)	8 (24)		17 (57)
**Comorbidity (CCI)**				
< 5 points	164 (47)	9 (27)	**0.03**	10 (33)
≥ 5 points	183 (53)	24 (73)		20 (67)
**Localization**				
Perianal	46 (13)	22 (67)	**<0.0001**	16 (53)
Anal	301 (87)	11 (33)		14 (47)
**T stage**				
1–2	186 (53)	17 (52)	0.82	19 (63)
3–4	161 (47)	16 (48)		11 (37)
**N stage**				
N0	173 (50)	11 (32)	0.07	13 (43)
N+	174 (50)	22 (68)		17 (57)
**M stage**				
M 0	330 (95)	31 (94)	0.77	30 (100)
M 1	17 (5)	2 (6)		-
**Treatment schedule**				
CRT A (44 Gy)	6 (3)	2 (6)		1
CRT B (54 Gy)	100 (29)	7 (22)	0.35	7 (23)
CRT C (58 Gy)	227 (68)	24 (72)		20 (67)
RT only (61–64 Gy)	14 (4)	-		3 (10)

CCI: Charlson Comorbidity Index; CRT: chemoradiotherapy; RT: radiotherapy.

*P*-values indicated are for the comparison between p16+ and p16- patients in the Uppsala cohort.

P-values in bold indicate statistical significance (p<0.05).

Following completion of treatment medical examinations were performed by ASCC experienced oncologists every 2 months during the initial 6 months, then every 3 months for the next 1.5 years, every 6 months during years 3 and 4 and then at a final visit at 5 years. Digital examination was performed at all clinical controls. MRI and PET-CT scans were performed 4 months after finished treatment. For patients with more advanced tumors and cCR at 6 months, CT-scans were done at 12, 24 and 36 months. In case of alarming symptoms or new lesions, biopsy and/or imaging examination were performed.

To compare and validate the findings from the Uppsala ASCC cohort, corresponding data were collected for p16- ASCC patients treated between 2014 and 2022 at the Sahlgrenska University Hospital in Gothenburg. Treatment and follow-up were similar for the Uppsala and Gothenburg cohorts with only minor differences in radiation doses, target delineation, and chemotherapy used for CRT.

### Statistical analysis

Categorical variables were described as frequencies or percentages with 95% confidence interval (CI) and were compared using Chi-square or Fisher’s exact tests. Survival analyses were performed using the Kaplan-Meier method with log-rank statistics. Variables with significant impact on outcome in univariable analysis were included in Cox multivariable analysis. *P*-values <0.05 were considered statistically significant.

Explorative analyses of prognostic factors in the Uppsala p16- patients for OS and persistent disease at 6 months were done using multivariable linear and logistic regression, respectively. GraphPad Prisma software (version 10 for Mac), was used for all statistical analyses.

## Ethical approval

The study was conducted in accordance with the general data protective regulation (GDPR) and approved by the Swedish Ethical Review Authority (registration numbers Dnr: 2022-01206-01 and 2023-02327-01).

## Results

### Patients and patient characteristics

The Uppsala cohort comprised a total of 415 patients. Thirty-five patients were excluded; 8 due to missing p16 status and 27, 6 of which p16-, who were treated with palliative intent, leaving 380 patients for further analysis. Median follow-up after the last day of CRT or RT was 33 months (range 4–78). Mean patient age was 67 years (range 29–89) and 289 (76%) were female (Table 1). Thirty-three (9%) patients had p16- tumors and 247 (65%) of patients were former or current smokers. The most frequent CCI scores were 4 or 5. There was no difference in smoking, tumor stage or treatment schedule between the p16+ and p16- groups. Age ≥ 65 years (*p* = 0.03), male sex (*p* = 0.0001), comorbidity (*p* = 0.03) and perianal tumor localization (*p* < 0.0001) were significantly more common in p16- compared with p16+ ASCC (Table 1).

The Gothenburg cohort comprised a total of 322 patients, 30 of which had p16- ASCC treated with curative intent and were included in the study to validate the findings for the Uppsala p16- patients. Median follow-up for the validation cohort was 28 months (range 5–91). The clinical characteristics of this p16- cohort were similar to the corresponding Uppsala cohort (Table 1).

### Outcome

**Figure 1:**
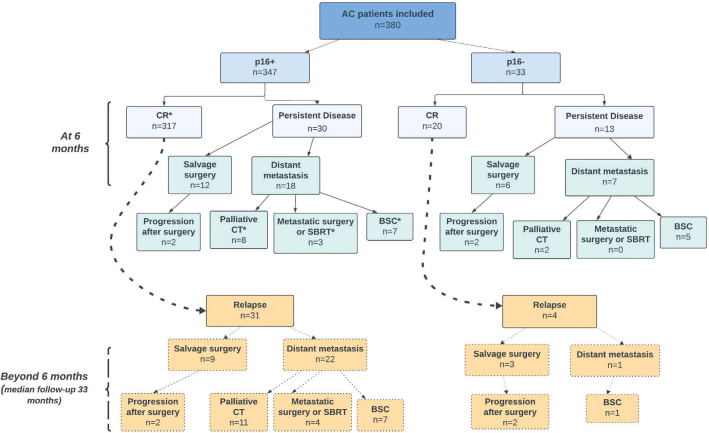
Overall treatment outcome according to p16-status for the Uppsala cohort (*n* = 380). See Table 2, for details on outcome and statistical inference.

At 6 months follow-up cCR was statistically significantly lower in p16- compared with p16+ patients, 61% versus 91% (*p* < 0.0001; Table 2). Persistent disease qualifying for salvage surgery was statistically significantly higher in p16- patients, 18% versus 3% (*p* < 0.0001). Development of distant metastasis within 6 months follow-up was significantly more common among p16- than p16+ patients, 21% versus 5% (*p* = 0.0030). Beyond 6 months local recurrence qualifying for salvage surgery was more common in p16- patients, 23% versus 3% (*p* = 0.0086). p16 status re-analyzed on surgical specimens after salvage surgery showed no change in p16 status compared with that at diagnosis (not shown). In p16- patients the cCR was observed in only 61% (*p* < 0.0001) with a 31% relapse rate among those who achieved cCR. Outcome over time is detailed in Figure 1 and Table 2.

**Table 2:** Outcome over time for the Uppsala cohort of p16+ and p16- patients.

Outcome	p16+ (*n* = 347)	p16- (*n* = 33)	*P*
**At 6 months**	***n* (%)**	***n* (%)**	
Complete remission	317 (91)	20 (61)	**< 0.0001**
Salvage surgery	12 (3)	6 (18)	**0.0024**
Distant metastasis	18 (5)	7 (21)	**0.0030**
Palliative CT	8 (2)	2 (6)	**0.0212**
Metastatic surgery or SBRT	3 (1)	-	> 0.999
BSC	7 (2)	5 (15)	**0.0019**
Progression after salvage surgery	2 (17)	2 (33)	0.0568
**Beyond 6 months**	(*n* = 317)	(*n* = 13)	
Relapse	31 (10)	4 (31)	0.0683
Salvage surgery	9 (3)	3 (23)	**0.0086**
Distant metastasis	22 (7)	1 (3)	> 0.999
Palliative CT	11 (3)	-	0.6175
Metastatic surgery or SBRT	4 (1)	-	> 0.999
BSC	7 (2)	1 (3)	0.2950
Progression after salvage surgery	2 (22)	2 (66)	0.2657

CT: chemotherapy; SBRT: stereotactic body radiotherapy; BSC: best supportive care.

P-values in bold indicate statistical significance (p<0.05).

**Figure 2:**
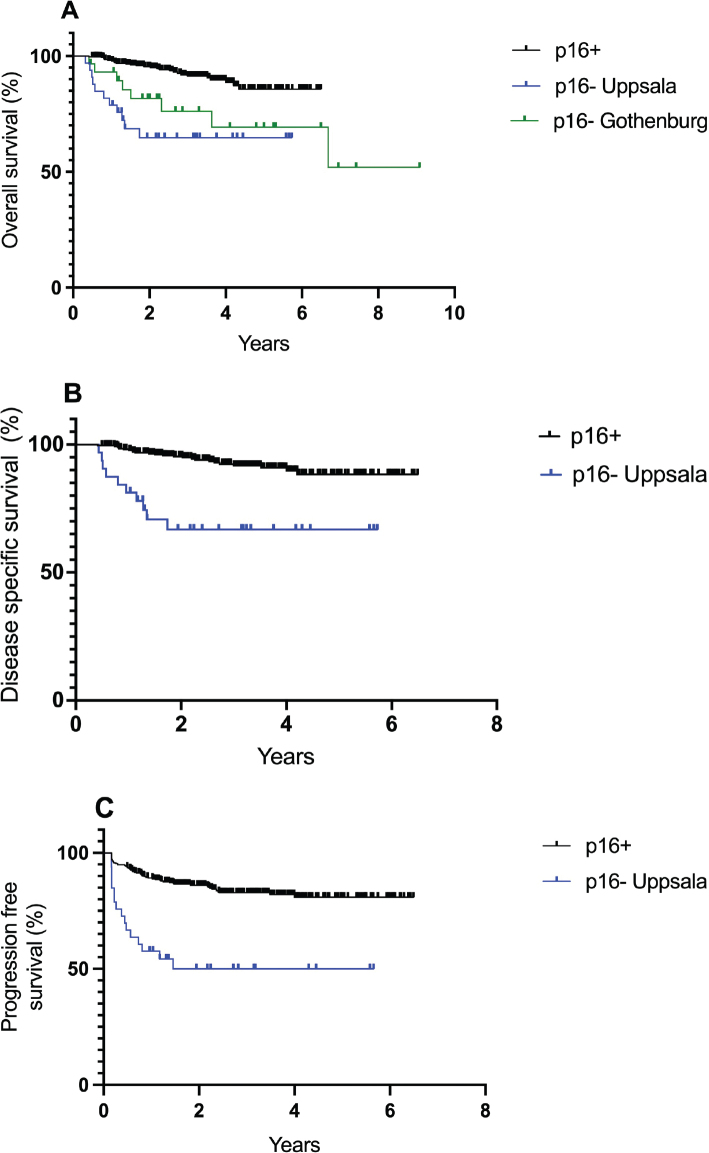
Overall survival according to p16-status with p16- patients separated for treatment center, Gothenburg cohort used as a validation cohort (a) and disease specific survival in Uppsala patients (b) and progression free survival (c) according to p16-status in Uppsala patients.

OS was significantly shorter in p16- compared with p16+ patients. At 3-years OS was 60% and 89% in p16- and p16+ patients, respectively (*p* = 0.0021) with a hazard ratio (HR) for death of 5.2 (95% CI: 1.5–18; logrank *p* < 0.0001; Figure 2a). Three-year DSS was lower in p16- compared with p16+ ASCC (HR: 5.3, 95% CI: 1.4–19.3; logrank *p* < 0.00013; Figure 2b). PFS at 3 years was 50% and 81% in p16- and p16+ patients, respectively (*p* = 0.0034) with a HR for progression of 4.1 (95% CI: 1.6–10.6; logrank *p* < 0.0001; Figure 2c).

Univariable analysis showed that male sex, advanced tumor stage (T3-4), N+ disease, treatment with schedule C and p16- were associated with inferior OS (*p* = 0.01 – *p* < 0.0001; Table 3). The multivariable analysis confirmed the negative prognostic impact of p16- (HR: 5.86, 95% CI: 2.5–12.6; *p* < 0.0001) and treatment with schedule C (HR: 5.6, 95% CI: 1.2–39.5; *p* = 0.040).

**Table 3:** Univariable and multivariable analysis of prognostic factors for overall survival in the Uppsala cohort.

Prognostic factor	Univariable				Multivariable		
	No	HR	95% CI	*P*	HR	95% CI	*P*
**Age (years)**				0.18			0.43
< 65	171	0.64	0.34 to 1.22		0.76	0.37 to 1.48	
≥ 65	209						
**Sex**				**0.01**			0.82
Male	91	2.2	1.0 to 4.68		0.91	0.40 to 1.93	
Female	289						
**Smoking**				0.16			
Yes	247	1.67	0.87 to 3.22				
No	133						
**CCI (points)**				0.14			
< 5	173	0.62	0.33 to 1.15				
≥ 5	207						
**Localization**				0.48			
Anal	68	0.74	0.29 to 1.85				
Perianal	312						
**T stage**				**0.0004**			0.096
1–2	203						
3–4	177	3.06	1.74 to 6.01		1.96	0.93 to 4.63	
**N stage**				**0.005**			0.91
N0	184	0.38	0.20 to 0.72		0.96	0.42 to 2.01	
N+	196						
**Treatment**				**<0.0001**			**0.040**
A + B	115	0.09	0.05 to 0.18		0.17	0.03 to 0.80	
C	251						
**p16 status**				**<0.0001**			**<0.0001**
+	347						
-	33	5.3	1.54 to 18.1		5.86	2.54 to 12.64	

HR: hazard ratio; CI: confidence interval; CCI: Charlson comorbidity index.

P-values in bold indicate statistical significance (p<0.05).

In explorative analyses in p16- patients to identify prognostic factors for OS low age (≤65 years) (HR: 0.51, 95% CI: 0.14–1.78; *p* = 0.036), lower tumor stage (HR: 0.16, 95% CI: 0.05–0.53; *p* = 0.007) and treatment schedule A or B (HR: 0, *p* = 0.009; Table 4) were associated with favorable outcome. No deaths were observed in patients treated with schedule A or B. However, none of the prognostic factors were statistically significantly associated with persistent disease at 6 months.

**Table 4:** Multivariable analysis of prognostic factors in the Uppsala p16- patients, (n = 33) for overall survival and for persistent disease at 6 months using multivariable linear and logistic regression, respectively.

Prognostic factor	Overall survival				Persistent disease		
	No	HR	95% CI	*P*	OR	95% CI	*P*
**Age (years)**							
< 65	9	0.51	0.14 to 1.78	0.36	0.96	0.85 to 1.08	0.59
≥ 65	24						
**Sex**							
Male	17	1.04	0.32 to 3.41	0.94	1.84	0.31 to 11.31	0.49
Female	16						
**Smoking**							
Yes	25	1.77	0.47 to 6.58	0.46	7.1	0.54 to 21.3	0.16
No	8						
**CCI (points)**							
< 5	9	0.57	0.15 to 2.13	0.47	1.06	0.06 to 18.2	0.96
≥ 5	24						
**Localization**							
Anal	11	0.60	0.08 to 4.16	0.65	0.71	0.09 to 4.89	0.72
Perianal	22						
**T stage**							
1–2	17	0.16	0.05 to 0.53	**0.007**		0.36 to 3.68	0.80
3–4	16						
**N stage**							
N0	11	0.42	0.12 to 1.44	0.25	0.27	0.01 to 4.02	0.35
N+	22						
**Treatment**							
A + B	9	0	-	**0.009**	1.09	0.001 to 50.5	0.90
C	24						

HR: hazard ratio; CI: confidence interval; OR: odds ratio; CCI: Charlson comorbidity index.

P-values in bold indicate statistical significance (p<0.05).

The outcome for the p16- Gothenburg cohort was comparable to that of the corresponding Uppsala cohort. At 6 months follow-up cCR was 63% with a relapse rate of 11% among those who achieved cCR at the 6-month follow-up. Persistent disease qualifying for salvage surgery was 23%. Three-year OS in p16- patients from Gothenburg was 64% (*p* = 0.004 vs. p16+ Uppsala) with an HR for death of 2.5 (95% CI: 1.1–5.5; logrank *p* = 0.0004; Figure 2a).

## Discussion

To our knowledge, our study presents data on one of the largest cohorts to date of ASCC patients treated in accordance with current guidelines. In agreement with previously published data, we observed a worse OS for p16- compared with p16+ [19, 32–35] ASCC patients.

As HPV vaccination programs are rolled out, HPV related malignancies are expected to decrease whereas HPV-negative cancers will be unaffected and probably represent a larger fraction of squamous cell malignancies. It is therefore important to gain knowledge which ultimately results in better treatment and outcome for this group of patients.

Our study adds to the knowledge of p16- ASCC with respect to its clinical characteristics and response to curative CRT. Patients with p16- ASCC were older, more often of male sex, had more comorbidity and presented more often with perianal localization. Furthermore, we found a higher rate of relapsed disease among cCR patients who were p16-negative. Moreover, patients with a p16- tumor had more frequently distant metastases at early follow-up and more frequently underwent salvage surgery than their p16+ counterparts.

This suggests that p16- ASCC constitutes a biologically more aggressive subset, often rendering patients unfit for further treatment. Within the p16- group patients high age, advanced tumor stage and treatment schedule C were significantly associated to inferior OS, as expected, whereas none of the prognostic factors were identified to predict for persistent disease at 6 months follow-up. Thus, we could not identify any clinical factor to be clearly decisive for response to CRT. Molecular pathological analysis of tumors from p16- patients with cCR compared with persistent disease may provide knowledge in features associated with CRT sensitivity.

Based on these clinical characteristics, p16- patients should be considered a separate ASCC entity requiring improved therapy, intensified and/or with components in addition to CRT, perhaps even upfront surgery. The underlying reason for the inferior survival in these patients remains unclear. While high age may preclude more aggressive treatments and advanced tumors are more therapy resistant and prone to metastasis, these factors alone do not fully account for the observed outcomes in p16- patients. Molecular and biomarker expression studies show that p16- ASCC more frequently has mutations in P53 [19, 26, 36] and CDKN2A [9, 21, 37] and overexpression of Epidermal growth factor receptor (EGFR) [20, 23] and programmed death ligand 1 (PD-L1). However, PD-L1 expression as a prognostic factor in ASCC is still unclear [38, 39].

On the other hand, a higher presence of CD3+, CD8+, PD-1+ [9, 25, 26], tumor infiltrating lymphocytes (TILs) [17, 26, 40] and high mutational tumor burden (TMB) [41] are associated with a better survival while PIK3CA mutations [36, 42] have been associated with worse survival in ASCC irrespective of p16 status. Adding analysis of molecular features in ASCC could possibly point to benefit from targeted therapies. Furthermore, p16 status combined with analysis of prognostic biomarkers could allow for a more individualized and/or stratified approach when it comes to follow-up after curative treatment. Depending on the tumor biomarker features some patients might need more intense and longer follow-up whereas for others it could be shortened and simplified.

Our study has strengths and limitations. Key strengths are cohort size, homogenous treatment in agreement with current guidelines within a limited period of time and validation of the findings for p16- patients with a similar cohort from another center. Furthermore, data on the specific clinical features of p16- ASCC add new knowledge to characterize this ASCC entity.

Among limitations are the retrospective study design, p16 analyses performed at different hospitals before patient referral and lack of centralized re-analysis of the p16 status. Furthermore, adding molecular pathology analysis, for example, using a next generation sequencing approach with a cancer specific gene panel could have added biological knowledge on the difference between p16+ and p16- ASCC. This approach is under consideration with focus on the p16- patients. The median follow-up time is just below 3 years. However, since ASCC relapses are very infrequent after 3 years [30], we believe that our data will not change considerably after longer follow-up.

## Conclusions

In conclusion, the present study adds insights in how p16- ASCC differs from p16+ disease in clinical features, treatment response and prognosis. Future studies should aim to explore more effective treatments for this poor-prognosis subgroup of ASCC.

## Supplementary Material

Clinical characteristics and treatment outcome in p16 negative anal cancer

## Data Availability

The data that support the findings of this study are available on request from the corresponding author. The data are not publicly available due to ethical restrictions protecting the privacy of the research participants.
