# Supplementary material for: Clinical characteristics and treatment outcome in p16 negative anal cancer
Source: Acta Oncol. 2025 May 7;64:42498. doi: 10.2340/1651-226X.2025.42498 (PMC12067988; doi:10.2340/1651-226X.2025.42498)
Supplement: Clinical characteristics and treatment outcome in p16 negative anal cancer [file AO-64-42498-s1.pdf]

Supplementary material has been published as submitted. It has not been copyedited, or typeset by Acta Oncologica

Appendix

Table 1.

Standardized treatment schedules following the National Swedish Care Program for Anal Cancer.

Treatment schedule

**TNM**	**Schedule**	**Chemotherapy**	**Radiotherapy**
T1N0M0	A	Cap* or 5-FU** + 1 MMC***	44 Gy to tumor<br>40 Gy to elective nodes
T1-2(<4 cm) N0M0	B	Cap or 5-FU + 1 MMC	54 Gy to tumor<br>40 Gy to elective nodes
T2 (>4cm)-T4N+M0	C	Cap or 5-FU + 2 MMC	58 Gy to tumor<br>58 Gy to N+ >2 cm<br>50 Gy to N+> 2 cm<br>40 Gy to elective nodes
Only RT			64-60 Gy to tumor<br>54 Gy to N+<br>46 Gy to elective nodes

*Capecitabine: 825 mg/m^2 twice daily, 5 days per week for all days of RT.
**5-fluorouracil: infusion dose 1 000 mg/m^2 days 1–4 and days 29–32.
***Mitomycin C: 10 mg/m2 once or twice, 1 MMC: day 1. 2 MMC: day 1 and 29.
